# Challenges of cellulitis in a lymphedematous extremity: a case report

**DOI:** 10.1186/1757-1626-2-9377

**Published:** 2009-12-22

**Authors:** Matthew Pierce Connor, Richard Gamelli

**Affiliations:** 1 Loyola University of Chicago, Stritch School of Medicine, 2160 South First Ave, Maywood IL, 60153, USA

## Abstract

**Introduction:**

Lymphedema is a relatively common phenomenon. It is important that clinicians appreciate the relative risks imposed by this condition. While for some it may only represent a flaw in appearance, this condition can potentially have fatal consequences. Our case reports on the challenges of cellulitis in a lymphedematous extremity that progressed to septic shock.

**Case presentation:**

A 37-year-old Hispanic male was transferred to the Burn Unit from an outside hospital for wound care of an extremely severe case of cellulitis. He suffered massive lymphedema of his lower extremity, with innumerable nodules and chronic skin changes. After 3 days of cellulitis, he was in critical condition and required intubation and vasopressors. With intense wound care and systemic antibiotics, he gradually recovered and was discharged in 16 days with his cellulitis resolved and ambulating independently.

**Conclusion:**

Our case highlights the special care and attention that chronic lymphedema deserves. These patients can exhibit marked disfigurement and physical disability affecting them on both social and physical levels. They also are at great medical risk, as cellulitis almost cost our patient his life. Evidence indicates that lymphedema, no matter the etiology, is susceptible to cellulitis with both great propensity and virulence. Physicians should be aware of the great risk of lymphedema, strive to prevent deterioration and complications, and be prepared to educate and closely monitor these patients.

## Introduction

No matter the etiology, patients with lymphedema commonly suffer from cellulitis. Such swollen skin is at high risk for breakdown and subsequent infection. Additionally, the hyperkeratotic and papillomatous surface harbors microbes, particularly when deep folds and crevices are present [1]. Lymphedema has even been documented as the most important risk factor for cellulitis [2]. When infection ensues in this environment, these patients are left with a compromised immune system. The stagnation of lymph limits the clearance of bacteria. Their cell-mediated immunity is upset as lymphatic travel of host immune cells is disrupted [3]. As a consequence, these patients are unable to mount an effective defense against pathogens. Not only do they suffer recurrent cellulitis, but they also experience prolonged bouts of cellulitis with longer and more intense systemic inflammatory responses [4]. This phenomenon is a direct result from their inability to clear bacteria, leading to continued cytokine release and inflammation. Persistent inflammation, in turn, worsens swelling and adds further damage to their lymphatic system placing the patient in a vicious cycle.

## Case presentation

A 37-year-old Hispanic male presented to the Loyola University Medical Center Burn Unit after being transferred from an outside hospital with concern for necrotizing fasciitis. This man suffered from chronic, painless lymphedema of his right lower extremity for the past 10 years. His lymphedema had accelerated in recent months, and his leg started to accumulate numerous nodules and verrucae. He began having intense pain in his affected leg and swelling in his scrotum. He reported having fevers, chills, diarrhea, and persistent nausea and vomiting for three days prior to being brought to an outside hospital. He presented to their emergency room delirious, hypotensive, febrile, tachycardic, and in respiratory distress meeting the requirements for systemic inflammatory response syndrome. Erythema encompassed his entire right leg and spread across his genitalia and to his left groin. Concern for Fournier's gangrene brought him to the OR for an exploratory scrotal operation. Fournier's was ruled out, and his incision was left open to heal by secondary intention. His condition continued to worsen, and was subsequently transferred to Loyola intubated and maintained on continuous norepinephrine.

**Figure 1:**
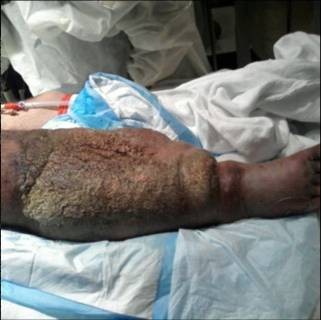
**The patient at presentation**. Figure illustrate the massive edema and erythema present, especially when compared to the contralateral leg.

**Figure 2:**
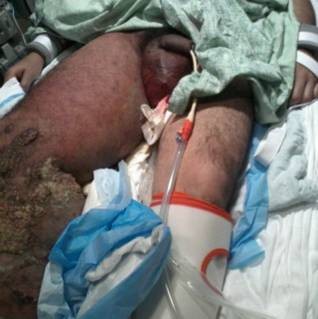
**Massive edema and erythema extended to his upper leg and across his genitalia**.

**Figure 3:**
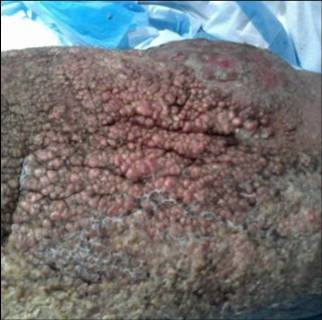
**Verrucae and nodularity encompassed his entire extremity**.

On initial exam, he was hypotensive at 94/32 mmHg, tachycardic at 132 beats/min, saturating 95% O2 on SIMV, and with a fever of 40.1°C. Arterial blood gasses demonstrated an anion gap metabolic acidosis. His leg was erythematous and hot to the touch, with blistering and pustular exudates. His scrotum and penis were markedly swollen and erythematous, and serous drainage came from his midline scrotal incision (Figures 1, 2, 3).

The working diagnosis was cellulitis superimposed on elephantiasis that led to a state of septic shock. His wounds were managed as per partial thickness burns care with twice daily debridement and application of silvadene and kerlix. His antibiotic regimen began with high dose penicillin drip, ciprofloxacin, and clindamycin for broad base coverage. His coverage was altered as cultures returned from both the outside hospital and Loyola. His leg wound cultures grew Proteus (pan-sensitive), MSSA, coagulase-negative Staphylococcus, and Candida parapsilosis. His scrotum wound cultures grew Group G Streptococcus, Peptostreptococcus magnus, and anaerobic gram negative rods that were not able to be identified (Bacteroides fragilis was excluded). His blood, urine, and BAL cultures had no growth. A microfilariae test for bloodborne parasites was negative. Given the anaerobes and yeast involved, his antibiotic regimen was changed to fluconazole, metronidazole, and ampicillin/sulbactam. He spiked fevers through the first week of his hospital stay, but this resolved and the erythema on his leg gradually improved with continual systemic and topical antibiotics.

He was maintained on norepinephrine for the first two days to keep his mean arterial pressure above 60 mmHg, then was successfully weaned and hemodynamically stable for the remainder of his hospitalization. The patient was successfully extubated on hospital day 7, and began breathing on room air with good oxygen saturation on day 8. His case was complicated by acute renal insufficiency. He presented oliguric with rising BUN and Creatinine. Fractional excretion of urinary sodium was 10%, and gross blood and small protein was seen in his urinalysis all factors leading to a diagnosis of acute tubular necrosis secondary to ischemia suffered in septic shock. His acidosis was treated with serial amps of bicarbonate plus maintenance fluids of 1 liter 5% dextrose with 80 ml Sodium Acetate. While his acidosis steadily resolved and his urine output rose, his BUN and Creatinine continue to rise reaching a level of 117 and 8.27, respectively. He received dialysis on Loyola hospital day 4, and received 3 additional dialysis treatments over the next 7 days. After these treatments his electrolyte levels stabilized, and he began having large urine output indicative of post-ATN diuresis.

Additional therapy included nutritional support with 1/2 strength Novasource renal tube feeds at 100 cc/hr, glucose control with insulin lispro sliding scale, and physical therapy. He was malnourished at presentation (Albumin 1.4 gm/dl, Transferrin 99 mg/dl), but improved with continual tube feeds and general diet. The patient was discharged on hospital day 16 with his cellulitis resolved, renal function and electrolyte levels normalized, ambulating independently with physical therapy, and tolerating a general diet. His lymphedema had actually improved somewhat since admission, but he maintained significant edema in the extremity. He subsequently followed up in the burn clinic 2 weeks later without any signs of infection but still with difficulty ambulating secondary to his condition.

## Discussion

The etiology of our patient's lymphedema is uncertain. Lymphedema is typically classified as either primary or secondary. The primary causes result from an inherent defect in the lymphatic channels. They can organized by age of onset and include congenital lymphedema (age <1 year), lymphedema praecox (age 1-35), and lymphedema tarda (age <35 years) [5]. Congenital lymphedema can be either familial or sporadic. The familial form is termed Milroy's disease and represents an autosomal dominant disorder thought to be due to a defect in the VEGFR-3 gene [6]. These patients have absent lymphatic channels in one or more extremities. Congenital lymphedema can also be found as part of Turner and Noonan syndromes. Lymphedema praecox, also known as Meige's disease, is the most common form of primary lymphedema. Onset is usually at puberty, and it is commonly associated with other anomalies like extra eyelashes (distichiasis). Histologically, these patients have hypoplastic lymphatic channels that may be a result of mutations traced to the FOXC2 gene [5,7]. Lymphedema tarda usually presents in adult patients with a family history of lymphedema. They have a congenital weakening of their lymphatics so that, with precipitating trauma, they eventually deteriorate and acquire lymphedema (5). Our patient did not have a family history of lymphedema, and his age of onset (27) and lack of other associated anomalies does not perfectly fit the profile for congenital, praecox, or tarda lymphedema.

The known secondary causes of lymphedema include malignancy, recurrent cellulitis, contact dermatitis, trauma, and filariasis. In the industrialized world, the most common etiology is malignancy and associated operative trauma [5]. However, the most common cause in the developing world is filariasis, as it affects over 120 million people in 80 countries worldwide [8]. In this mosquito-borne parasitic infection, microscopic worms live in the human lymph system and disrupt normal lymph flow. Workup for a secondary cause of lymphedema in our patient was negative. He did not have a history of malignancy, recurrent cellulitis/dermatitis, or trauma to his extremity. The possibility of filariasis is unlikely given he had never been outside of Mexico or the US. According to the CDC, lymphatic filariasis cannot be contracted in the US, and no reports of this parasitic infection have been recently documented in Mexico. The parasite is most common in Asia and Africa, but is present in parts of the Caribbean and South America. To document infection, a blood smear test should be performed between 10 pm and 2 a.m. for the best chance to detect microfilariae (termed "nocturnal periodicity") [9]. However, because lymphedema may develop many years after infection, lab tests are often negative.

Given this background, our patient most likely suffered from a primary lymphedema and may represent a variant of the known syndromes. The only medical treatment he ever received was in the form of a leg stocking given to him by a local physician in Mexico, which was of marginal benefit. He was never given an explanation for his lymphedema, and was understandably apprehensive about his future health.

## Conclusion

To prevent episodes of such severe cellulitis like our patient experienced, it is recommended that these patients seek medical care immediately after symptoms present. A self-medication strategy has even been proposed to ensure quick treatment and prevent the kind of complications that our patient experienced [9]. In this scenario, the patient is supplied with antibiotics to independently use at the first sign of cellulitis. No reports of such a strategy have thus far been studied, but could very well deserve attention. In cases of recurrent infection, prophylactic phenoxymethyl penicillin or cephalexin are recommended. Salicylic acid ointment can also be used to help control skin surface microbe colonization [5]. Aims to reduce existing lymphedema try to channel the fluid into surrounding, intact lymphatic vessels. Manual lymph drainage, compressive bandaging, and specific physical therapy exercises can be successful, but it is unlikely that these therapies would be useful in such a severe case as ours. Surgery can be reconstructive through creation of lymph-venous shunts or via autologous vessel transplantations. Surgical debulking of excess tissue can be tried to improve mobility and function. However, wound healing complications are prevalent in these cases [5].

One other case report found in the literature similarly described cellulitis in a lymphedematous lower extremity that led to systemic inflammatory response syndrome [10]. In this case, the etiology of lymphedema was oopherectomy and pelvic lymph node dissection for ovarian malignancy. This patient was actually successfully treated after debridement of her wound and placement of a split-thickness graft.

Our case highlights the special care and attention that chronic lymphedema deserves. These patients can exhibit marked disfigurement and physical disability affecting them on both social and physical levels. They also are at great medical risk, as cellulitis almost cost our patient his life. Evidence indicates that lymphedema, no matter the etiology, is susceptible to cellulitis with both great propensity and virulence. Physicians should be aware of the great risk of lymphedema, strive to prevent deterioration and complications, and be prepared to educate and closely monitor these patients.

## Competing interests

The authors declare that they have no competing interests.

## Authors' contributions

MC participated in the management of the case as well as reviewed the literature pertaining to lymphedema. MC is the first author of this case report, and received contributions from Dr. Richard Gamelli.

## Consent

Written informed consent was obtained from the patient for publication of this case report and accompanying images. A copy of the written consent is available for review by the Editor-in-Chief of this journal.
